# Anti-tumorigenic Effects of Emodin and Its’ Homologue BTB14431 on Vascularized Colonic Cancer in a Rat Model

**DOI:** 10.31557/APJCP.2020.21.1.205

**Published:** 2020

**Authors:** Philipp Höhn, Chris Braumann, Maria Freiburger, Gerold Koplin, Wolfgang Dubiel, Andreas Minh Luu

**Affiliations:** 1 *Department of General and Visceral Surgery, Division of Molecular and Clinical Research St. Josef-Hospital, Ruhr-University Bochum, Bochum, *; 2 *Private veterinary practice of Maria Freiburger, Lehesten, *; 3 *Clinic for Minimal Invasive Surgery (MIC), Berlin, *; 5 *Institute of Experimental Internal Medicine, Medical Faculty, Otto von Guericke University, Leipziger Str. 44, 39120 Magdeburg, Germany, *; 4 *School of Pharmaceutical Sciences, Xiamen University, Xiang’an South Road, Xiamen 361102, China. *

**Keywords:** Colon cancer, emodin, BTB 14331, rat model

## Abstract

**Objective:**

New drugs for cancer treatment are being sought worldwide. Therapeutic agents derived from natural substances can provide cost-efficient options. We evaluated the effect of emodin, an active natural anthraquinone derivate, and it’s in-silico homologue the novel substance BTB14431 in vivo.

**Method:**

CC-531 colon cancer cells were implanted intraperitoneal (ip) and subcutaneous (sc) in 100 WAG/Rij rats. 28 days after tumor cell implantation, solid cancers were treated for 7 days by varying doses of BTB14431 (0.3 mg/kg body weight; 1.7 mg/kg) or emodin (2.5 mg/kg; 5 mg/kg). Treatment was applied either via an intravenous (iv) port catheter or by ip injection. Saline solution served as control. 21 days after final dose all animals were euthanized and ip tumor weight, sc tumor weight and animal body weight (bw) were determined by autopsy. Significant lower total tumor weight occurred after iv treatment with low dose BTB14431 (6.8 g; 90% confidence interval (CI) 5.3 - 8.2 g; p ≤ 0.01) and also low and high concentrations of emodin (9.4 g; CI 7.9 - 10.7 g; p ≤ 0.01 and 8.3 g; CI 7.6 - 9.3; p ≤ 0.01). Iv treatment by high dose BTB14431 did not lead to a decline in tumor weight. High dose ip treatment by emodin led to a lower overall (11.1 g; CI 10.1 – 13.8 g; p ≤ 0.01) and ip tumor weight (8.6 g; CI 6 – 10.4 g; p ≤ 0.01). Sc tumor weight was not affected. All other ip treatments did not result in changes of combined, ip or sc tumor weight. Bw decreased during iv treatment in all animals and increased after treatment was completed. Regain of bw was stronger in animals receiving low dose emodin.

**Conclusion:**

Our study shows promising anti-cancer properties of BTB14431 and supports the evidence regarding emodin as a natural antitumorigenic agent. Optimal dosing of iv emodin and especially BTB 14431 for maximal efficacy remains unclear and should be a subject of further research.

## Introduction

Accounting for 1.2 million new cases globally each year, colorectal cancer is the third most Common type of cancer. It causes approximately 600.000 deaths per year being the third most common cancer cause of death. Neoadjuvant and adjuvant chemotherapy plays a major role in advanced stages of the disease (Bray et al., 2018). Even though advantages in survival have been achieved, development of new effective therapeutic agents remains important. Therapeutic agents derived from natural substances allow for cost-efficient availability as treatment costs generally rose in the last years (Fakih, 2015).

Emodin is an active natural anthraquinone derivate found in roots and rhizome of numerous Chinese medicinal herbs. In vitro anticancer properties have been demonstrated for multiple human cancer cell lines but research on colorectal carcinoma is still limited (Wei et al., 2013). The new substance BTB 14431 is an in-silico screening analog to emodin and was recommended by our former colleague Prof. Dubiel from Charité, Berlin, Germany. In vitro experiments showed stronger anti-tumorigenic capabilities against HeLa cervix carcinoma cells compared to emodin (Füllbeck et al., 2005). Our preliminary in vivo study confirmed tumor apoptosis under intraperitoneal (ip) and intravenous (iv) treatment with emodin and BTB 14431 but did not reveal a tumor suppressing effect in non-vascularized tumors (Braumann et al., 2017). As treatment effectiveness can be highly dependent on dose and protocol, the aim of our study was to establish an effective treatment protocol and compare effects on tumor mass and animal bw for ip and iv treatment with Emodin and BTB 14431.

## Materials and Methods

*Animals*

As in our previous study, we conducted the experiment with WAG/Rij rats (Charles River, Sulzfeld, Germany). A total of 100 animals were acquired. Permission was obtained by the local authorities (Landesamt für Gesundheit und Soziales, Berlin, Germany, CNoG0011/09) and strictly followed national animal protection laws. Animals were kept at 22-24^o^C and 50-60 % air humidity. Water and standard laboratory food were provided ad libitum. They were acclimated to a 12-hour light-cycle controlled environment 7 days before any procedures. Rats used were approximately 5 weeks of age and weighted 150 - 200 g. Animals were sacrificed 8 weeks after initial surgery via carbon dioxide (CO_2_) inhalation. 

*Tumor cell line*

CC-531 (received from the Deutsches Krebsforschungszentrum, Heidelberg, Germany) is a chemically induced colon adenocarcinoma cell line of the WAG-Rij rat. Cells were cultivated in standardized and sterile fashion (37^o^C, 5.2% carbon dioxide, and 94.8% oxygen) in RPMI-1640 medium (Merck, Berlin, Germany) supplemented with 10% heat inactivated fetal bovine serum (Gibco BRL, Karlsruhe, Germany), 2 mmol/l of glutamine (Biochrom, Germany) and 1000 IU/ml of penicillin/ streptomycin (Gibco BRL, Karlsruhe, Germany). Cells were harvested with EDTA and then washed three times in PBS (both from Gibco BRL, Karlsruhe, Germany) by centrifugation (Hereaus- Sepatech Varifuge 3.0 R; Kendro Hereaus, Burladingen, Germany) at 1000 rpm at a temperature of 4^o^C for 5 min. Vitality was determined by the trypan blue method using a Neubauer counting chamber. Cells were suspended in a serum-free medium to the desired concentration of 10^5^ Cells/ml and kept at 5% CO_2_ and 37°C until application. 

*Therapy agents*

Emodin (ACROS Organics, Geel, Belgium) and BTB 14431 (Sigma-Aldrich, Steinheim, Germany) were suspended in 5 % solution of polyvinylpyrrolidone (PVP, Sigma-Aldrich, Steinheim, Germany) and 0.9 % sodium chloride solution (Braun, Germany) according to target concentration in sterile fashion. Concentrations were chosen as 0.3 mg/kg bw and 1.7 mg/kg bw for BTB 14431 and 2.5 mg/kg bw and 5 mg/kg bw based on previous experiments by Braumann et al., (2017). Physiological Ringer-Lactate-Solution (Braun, Germany) served as control. 

*Study design*

Animals were randomized into ten groups (Table 1: Group I – Group X) with respect to the therapeutic agent, dose and either ip or iv application. 

**Figure 1:**
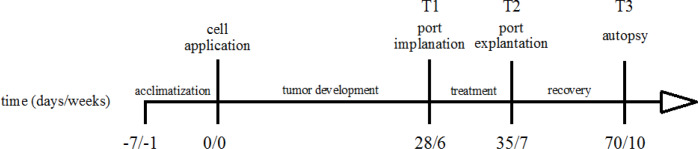
Timeline; Timeline of the Experiment, Indicating Time between Interventions

Surgical interventions were performed under general anesthesia using bw depended intramuscular injection of 12 mg/kg xylazine (Rompun, Bayer-AG, Germany) and 75 mg/kg s-ketamine (Ketanest, Pfizer, USA). After median laparotomy (4 cm) cecum and the small intestine were exenterated for 5 min. 1 ml of tumor cell suspension was administered into the abdominal cavity. Abdominal closure was performed by continuous suture (Vicryl 4-0, Ethicon). Immediately afterwards 1 ml of tumor cell suspension was injected sc into the neck of each animal. Figure 1 shows the timeline of our study and indicates relevant interventions.

On the 28^th^ postoperative day (T1) animals of group I – V underwent implantation of an iv port catheter (ROP-3.5H, Norfolk-Access Technologies, USA) into the right external jugular vein. One day after administering the last dose of chemotherapy, the iv catheter was removed (T2). 

Animals were treated according to their group either by iv (group I – V) or ip (group VI - X) application of 1 ml therapeutic agent every 12 hours over the course of seven days, beginning on the day of port implantation (28th day after tumor implantation). Treatment was performed under general isoflurane anesthesia. General condition of the animals was checked before and after any treatment. Bw was measured after the final treatment. 

56 days after tumor implantation (21 days after final dose of therapeutic agent) all animals were euthanized with carbon dioxide poisoning under general anesthesia (T3). The autopsy was performed by two assessors. The investigators were ‘blinded’ for the treatment. Sc and ip tumor weight and animal bw were determined. 

*Statistics*

Statistical analysis was performed using SPSS 25.0 (SPSS Inc., Chicago, IL). We used the Kruskal–Wallis test for unequal distribution of tumor weight and single comparisons were performed by the Mann–Whitney test. P values < 0.05 were considered significant. If not indicated otherwise values are presented as median and 90 % confidence interval. Doses are shown as mg per kg bw.

## Results

Ninety-two animals survived the entire treatment period: One animal died during port implantation, two animals died during iv applications and five animals died without any clear cause during chemotherapy. Statistical analysis was therefore performed with n = 92. 

*Tumor weight*

Combined ip and sc tumor weight in the control group was 15.9 g (14.5 – 20 g; iv treatment) and 19.8 g (15.4 – 23.3 g; ip treatment). Tumor weights after treatment by BTB 14431 and emodin are presented in Table 2. Significant lower total, ip and sc tumor weight occurred in group I, III and IV. Iv treatment by high dose BTB14431 (group II) did not lead to a significant decline in tumor weight. 

High dose treatment by emodin (group IX) led to a significant lower overall (11.1 g; 10.1 – 13.8 g; p ≤ 0.01) and ip (8.6; 6 – 10.4 g; p ≤ 0.01) tumor weight. Sc tumor weight was not affected. All other treatments (group VI – VIII) did not result in significant changes of combined, ip or sc umor weight. 

We also compared iv and ip treatment regarding tumor weight. Iv treatment by low dose BTB14431 (group I) led to a significantly lower tumor weight compared to ip treatment (group VI). Even though sc and total tumor weight was lower after iv treatment by high dose emodin (group IV) compared to ip treatment (group IX), ip tumor weight was not affected by the application modality (Table 4).

*Body weight*

We compared the animals’ bw percentage between the beginning (T1) and at the end (T2) of the treatment, between the beginning of the treatment (T1) and autopsy (T3) and also between the end of the treatment (T2) and the autopsy (T3, Figure 1). Measurements were not corrected for tumor weight. 

Bw decreased during iv treatment (T1 – T2) regardless of therapeutic agent or dose in all animals (group I – V). None of these changes were significantly different compared to control (group V: - 5.2 %; -5.8 - -3.0 %). After treatment (port removal) bw of all iv treatment groups increased until autopsy including control (group V: 3.1 %; 1.6 – 3.5 %). Low dose iv treatment by emodin led to a higher increase in bw (group III: 5.0 %; 4.1 – 6.8 %; p ≤ 0.01). regain after treatment (T2 - T3) regain almost completely compensated the initial loss, leading to no significant change in bw between T1 and T3. 

During ip treatment (T1 – T2) bw remained almost unchanged. Only for low dose emodin treatment a significant increase in bw compared to control was noticed (group VIII: .4 %; 0.1 – 1.1 %; p = 0.035). In between T1 and T3 bw of the control group remained almost stable whereas all treatments led to an increase in bw. Most signifcant effects were observed for both doses of BTB 14431. Regarding the time in between T2 and T3 bw of all treatment groups except low dose emodin showed a significant gain in bw after port removal.

We compared changes in bw regarding the treatment. Between T1 and T3 iv treatment by BTB 14331 resulted in a significant weight loss compared to ip treatment (group I vs group VI: p ≤ 0.01; group II vs group VII: p = 0.028). Comparison for T1 until T2 showed a significantly greater weight loss under iv treatment for control and all treatment groups (group I to V vs group VI to X: all p ≤ 0.01). Finally, for T2 to T3 increase in bw was significant higher for all iv treatment groups compared to ip treatment except for high dose BTB 14431 treatment (group VII vs group II: p = 0.243; other groups: p ≤ 0.01 to 0.025).

**Table 1:** Treatment Groups

	BTB14431		Emodin		Control (Saline)
	0.3 mg/kg	1.7 mg/kg	2.5 mg/kg	5 mg/kg	
iv treatment	Group I	Group II	Group III	Group IV	Group V
ip treatment	Group VI	Group VII	Group VIII	Group IX	Group X

Treatment groups as formed by agent and dose

**Table 2:** Tumor Weight Resulting from Different Treatment Modalities and Applied Doses

	BTB14431				Emodin				Control
iv treatment	0.3 mg/kg	I	1.7 mg/kg	II	2.5 mg/kg	III	5 mg/kg	IV	V
sc tumor weight (g)	2.5 (1.7 – 3.1) p = 0.035		3.7 (3.2 – 4.1) p = 0.604		2.2 (1.4 – 2.6) p ≤ 0.01		2.2 (1.5 – 2.7) p = 0.023		4.2 (3 – 4.7)
ip tumor weight (g)	4.5 (3.3 – 5.4) p ≤ 0.01		14.1 (12.7 – 16.8) p = 0.447		7.4 (6.3 – 8.3) p ≤ 0.01		6.4 (5.5 – 7.1) p ≤ 0.01		13.5 (11.1 – 15.8)
Total tumor weight (g)	6.8 (5.3 – 8.2) p ≤ 0.01		18.2 (16.0 – 20.8) p = 0.497		9.4 (7.9 – 10.7) p ≤ 0.01		8.3 (7.6 – 9.3) p ≤ 0.01		15.9 (14.5 - 20)
ip treatment	0,3 mg/kg	VI	1,7 mg/kg	VII	2,5 mg/kg	VIII	5 mg/kg	IX	X
sc tumor weight (g)	4.1 (2.7 – 4.8) p = 0.829		3.6 (2.5 – 4.7) p = 1.000		4.1 (3.2 - 5) p = 0.447		3.5 (2.8 – 4.7) p = 0.971		4.0 (2.6 – 4.5)


ip tumor weight (g)	9.0 (7.4 – 13.1) p = 0.055		11.8 (8.4 – 15.2) p = 0.156		11.3 (8.8 – 13.8) p = 0.079		8.6 (6 – 10.4) p ≤ 0.01		15.5 (12.1 – 19.5)


Total tumor weight (g)	13.3 (10.4 – 17.6) p = 0.101		14.3 (11.5 – 19.3) p = 0.243		15.8 (12.7 – 18.1) p = 0.243		11.1 (10.1 – 13.8) p ≤ 0.01		19.8 (15.4 – 23.3)



Subcutaneous (sc), intraperitoneal (ip) and overall median tumor weight at T3 after either intravenous (iv) or intraperitoneal (ip) treatment by varying doses of emodin, BTB14431 and electrolyte solution. Parentheses show the 90% confidence interval. P-value was obtained by Mann-Whitney-U-Test comparison against saline solution (control). Gray background color marks p values > 0.05.

**Table 4:** Comparison of Tumor Weight Regarding Treatment Modalities

	BTB14431				Emodin				Control
	0.3 mg/kg	I vs VI	1.7 mg/kg	II vs VII	2.5 mg/kg	III vs VIII	5 mg/kg	IV vs IX	V vs X
sc tumor weight	p = 0.043		p = 0.720		p ≤ 0.01		p = 0.033		p = 0.661
ip tumor weight	p ≤ 0.01		p = 0.095		p = 0.01		p = 0.230		p = 0.447
Total tumor weight	p ≤ 0.01		p = 0.182		p ≤ 0.01		p ≤ 0.01		p = 0.661

Comparison of subcutaneous (sc), intraperitoneal (ia) and overall median tumor weight at T3 after either intravenous (iv) or intraperitoneal (ip) treatment by varying doses of emodin, BTB14431 and electrolyte solution. Parentheses show the 90% confidence interval. P-value was obtained by Mann-Whitney-U-Test comparison of iv vs. ip therapy. Gray background color marks p values > .05.

## Discussion

Emodin is isolated from the root and rhizomes of Rheum palmatum L. and an active component in traditional Chinese and Japanese medicine. In-vitro and in-vivo anticancer properties of emodin have been demonstrated for multiple carcinoma types. These include direct induction of apoptosis and sensitizing tumor cells against other chemotherapeutic agents (Huang et al., 2007; Wei et al., 2013). In vitro research on colon carcinoma cells include HCT116, HCT–15 and PLR-3-expressed DLD-1 colon tumor cells. Emodin was found to cause direct apoptosis and reducing tumor cell invasiveness in these cell lines (Han et al., 2012; Xie et al., 2014). Regarding in-vivo models of colon carcinoma cells, the body of available literature is much more limited. Ma et al. examined sc injected LS1034 colon carcinoma cells in BALB/c nu/nu mice. Animals were treated by ip injection of 40 mg/kg every 3 d after tumor volume reached 200 mm³. Treatment by emodin resulted in a significantly lower tumor weight and smaller tumor volume after 39 days compared to 1 % DMSO. Results were similar to treatment by 5-FU (33 mg/kg) (Ma et al., 2012).

Using in-silico screening, a 3D superposition algorithm to compare computed molecular structures, BTB 14431 was identified as a chemical similar compound to emodin. In vitro testing on HeLa cervix carcinoma cells confirmed the induction of apoptosis by inhibiting CSN-associated kinases by BTB 14431. Observed effects were even stronger than for the lead substance (emodin) (Füllbeck et al., 2005).

Following up on the promising results of the in-silico screening, our research group performed a preliminary in vivo study on the effectiveness of emodin and BTB 14431 in treating CC531 colon carcinoma cells. 10^5^ Cells were applied ip and sc to WAG-RiJ rats. 120 animals were treated by different doses of emodin and BTB 14431. Sodium solution served as a control. Similar to our previous study, treatment was carried out either ip or iv via a central venous port catheter. Treatment was carried out every 12 h for 7 days. Animal bw was measured four times: 7 days before tumor implantation, at the start of treatment, at the end of treatment and 21 days after treatment. Following the last measurement, all animals were euthanized, and tumor weight was determined by autopsy. We also performed a hemogram and evaluated the laparotomy wound by histological examination. 

Significant reduction of ip tumor weight was achieved by iv treatment with BTB 14431 in a dose of 0.3 mg/kg. Sc tumor weight was not significantly affected. No other treatment protocols led to a significant difference compared to control. All animals receiving high dose iv emodin (10 mg/kg) did not survive treatment. Furthermore, sc and ip tumor weight was generally much lower in the ip treatment group compared to iv treatment including controls. However, wound healing was neither impaired by emodin nor by BTB 14431 in any treatment group compared to control. 

As other doses of BTB 14431 have not been studied, the optimal treatment concentration of BTB 14431 remains unclear. Iv treatment by concentrations between 0.3 mg/kg and 1.7 mg/kg might therefore be a promising subject for further research. This also includes the physiological causes of this relatively small “therapeutic window”. It is currently unknown whether ip treatment by BTB 14431 simply requires higher doses, as it might be the case for emodin (see below) or whether ip treatment by BTB 14431 is ineffective based on other mechanisms. As earlier research did not show toxic effects of ip treatment with 3.3 mg/kg, higher doses of BTB 14431 should be a subject of further research (Braumann et al., 2017).

Antitumorigenic capabilities of emodin also have been demonstrated for multiple other cancer cell lines in vivo and in vitro. 

Qu et al., (2015) performed a single intramuscular application of 300 µg/kg emodin combined with 800 ng High-Mobility-Group-Protein B1 (HMBG1), followed by daily ip application of the same dose for 14 days in male BALB/c nude mice. Effects on multiple sc implanted human osteosarcoma cell lines were evaluated. Emodin dramatically reduced HMGB1 induced VEGF production leading to a reduced tumor growth.

Intraperitoneal emodin-application of 40 mg/kg every other day for two months in athymic nude mice after injection of MDA-MB-231 human breast cancer cells caused a lower number of intrapulmonal nodules and a lower overall tumor weight. Animal bw and serum levels of aspartate aminotransferase, alanine aminotransferase and blood urea nitrogen did not differ compared to placebo (Sun et al., 2015).

Research by Iwanowycz et al., (2016) also concerning human breast cancer cells (EO771 and 4T1) showed that treatment of C57BI/6 or Balb/c mice by 40 mg/kg emodin ip once daily directly after tumor cell injection caused an inhibition of primary tumor growth. It reduced tumor size and tumor weight. Metastatic lung nodules caused by EO771 occurred significantly lower.

Liu et al., (2012) applied varying ip doses of emodin (10, 20, 40 or 80 mg/kg bw every 3 days) to BALB/c (nu/nu) mice a total of 5 times. Treatment began three weeks after peripancreatic implantation of 5x106 Panc-1 cells. They found an increased mice bw and decreased maximal tumor diameter correlating with higher doses. Treatment by 40 mg/kg resulted in a higher bw and equal tumor size compared to 80 mg/kg, being the most effective dose. They also observed increased food intake in the 80 and 40 mg/kg treatment groups.

Even though numerous studies regarding ip application of emodin exist, different treatment protocols hamper comparability. Treatment by up to 40 mg/kg daily has been shown to decrease tumor growth without significant side effects. Whether daily treatment with higher doses provides further benefits remains unclear. The role of different tumor stages (vascularized vs non-vascularized) leading to a varying susceptibility towards emodin is also controversial discussed in the studies mentioned above.

An important difference between our study and previous research regarding emodin and BTB 14431 is that our previous research focused on treatment directly following tumor application (non-vascularized tumor model). In contrast to our recent study, tumors where neither solid nor vascularized. Tumor growth beyond a diameter of 2 mm requires effective angiogenesis (Naumov et al., 2006). In vivo research on HCT116 cells suggest an inhibition of tumor angiogenesis via blocking VEGF-receptor signaling (Lu et al., 2008). Our current study aligns with these results by demonstrating a higher effect of iv treatment by emodin and BTB 14431 in case of vascularized tumors. Even though research on the specific tumor inhibiting mechanisms of BTB 14431 is currently limited, anti-angiogenic capabilities are a possible explanation and should be a subject for further research.

Weight loss and slower increase in bw of animals undergoing chemotherapy is a common observation. Bw alteration is used to measure toxicity of therapeutic agents in animal models. Weight loss can be as high as 20 % of pretherapeutic bw with agents as oxaliplatin (Hubert et al., 2015). In our study, a decline of bw while animals underwent iv treatment was observed. This effect occurred regardless of treatment agent or control, resulting in a loss of approximately 5 % of bw. It regenerated in all groups after treatment, leading to only minimal bw loss at obduction. We therefore attributed bw changes in the iv treatment group to an increased stress-level caused by the treatment itself. This can be a sign of emodin and BTB 14431 being of lower toxicity as comparable chemotherapeutic agents. Another possible interpretation is a protective capacity of emodin and BTB 14431 against cachexia. Further support of this argument can be found in other studies, showing an increased bw in animal models after treatment by emodin (Cha et al., 2005). 

In contrast, ip application did not lead to a decline of bw. We attribute the stable bw under ip treatment as a sign of lower metabolic stress as animals did not receive general anesthesia in contrast to iv treatment. Unfortunately considering the difference in tumor weight ip treatment might not be as effective as iv treatment. Therefore, gains in bw could be caused by increased tumor mass in the ip treatment group. 

Our study currently the only available research regarding animal’s bw after ip treatment by emodin or BTB 14431. While bw changes caused by iv treatment can be interpreted as a sign of efficacy and low toxicity these features remain at least questionable for ip treatment.

In conclusion, emodin is an active natural anthraquinone derivate and already showed anti-tumorigenic capabilities against various cancer cell lines in vivo and in vitro. Our study shows significant tumor weight reduction of ip and sc CC-531 tumors in WAG-RiJ rats after iv treatment by 2.5 and 5 mg/kg emodin without relevant toxicity. Similar effects could be demonstrated for treatment by 0.3 mg/kg BTB 14431. This substance is an in-silico homologue to emodin. Even though research on this substance remains limited, it showed promising anti-cancer activities. Optimal dosing of iv emodin and especially BTB 14431 for maximal efficacy remains unclear and should be a subject of further research. Other groups did achieve anti-tumor effects by applying high doses of emodin ip. These effects could not be observed (using a lower dose). Ip treatment with BTB 14431 did not result in lower tumor growth in rats. 
